# Oncostatin M Mediates Adipocyte Expression and Secretion of Stromal-Derived Factor 1

**DOI:** 10.3390/biology8010019

**Published:** 2019-03-23

**Authors:** Hardy Hang, Jennifer L. Bailey, Carrie M. Elks

**Affiliations:** 1 Adipocyte Biology Laboratory, Pennington Biomedical Research Center, Baton Rouge, LA, 70808, USA; hardy.hang@pbrc.edu; 2 Matrix Biology Laboratory, Pennington Biomedical Research Center, Baton Rouge, LA, 70808, USA; jennifer.bailey@pbrc.edu

**Keywords:** oncostatin M, adipose tissue, stromal-derived factor 1, adipocyte, inflammation

## Abstract

Adipose tissue homeostasis depends on interactions between stromal cells, adipocytes, and the cytokines and chemokines they produce. The gp130 cytokine, oncostatin M (OSM), plays a role in adipose tissue homeostasis. Mice, lacking the OSM receptor (OSMR) in adipocytes (*Osmr*^FKO^ mice), exhibit derangements in adipose tissue, insulin sensitivity, and immune cell balance. Here, we describe a possible role for the chemokine stromal-derived factor 1 (SDF-1) in these alterations. We treated 3T3-L1 adipocytes with OSM and observed a suppression of SDF-1 gene expression and protein secretion, an effect which was partially blunted by OSMR knockdown. However, *Osmr*^FKO^ mice also exhibited decreased SDF-1 gene and protein expression in adipose tissue. These contrasting results suggest that the loss of adipocyte OSM–OSMR signaling in vivo may be indirectly affecting adipokine production and secretion by altering OSM target genes to ultimately decrease SDF-1 expression in the *Osmr*^FKO^ mouse. We conclude that adipocyte OSM–OSMR signaling plays a role in adipose tissue SDF-1 production and may mitigate its effects on adipose tissue homeostasis.

## 1. Introduction

Interactions between stromal vascular cells (SVCs) and adipocytes are crucial for the maintenance of white adipose tissue (WAT) homeostasis. The chronic, low-grade inflammatory state associated with obesity is characterized by WAT leukocyte infiltration and alterations in the physiological cytokine milieu that favor pro-inflammatory adipokine production and disruption of the stem cell niche. These effects, in combination, can negatively affect overall WAT function and metabolism [1,2,3,4,5,6,7,8].

We have previously focused on the pro-inflammatory WAT adipokine, oncostatin M (OSM), and how its signaling may regulate tissue homeostasis and immune cell balance [9,10]. OSM belongs to the gp130 family of cytokines and regulates a variety of cellular and biological processes in a cell-type-dependent manner [11,12,13]. Unlike any other gp130 cytokine, OSM has its own specific receptor (oncostatin M receptor; OSMR) that heterodimerizes with gp130 to mediate OSM effects [14], which occur via JAK-STAT or ERK pathway activation [15,16,17,18,19,20]. We have demonstrated that, in WAT, SVCs—not adipocytes—produce OSM in conditions of obesity [9,21]. Another group has published data to indicate OSM can be expressed in adipocytes, but we are consistently unable to detect OSM in either cultured adipocytes or adipocytes isolated from mouse adipose tissue [9,21,22,23]. However, we and others have shown that adipocytes do express OSMR [9,10,21,24], which suggests that adipocyte OSM–OSMR signaling may play a role in WAT homeostasis.

We have generated mice with an adipocyte-specific deletion of OSMR (called *Osmr*^FKO^ mice) [9]. Chow-fed *Osmr*^FKO^ mice exhibit epididymal adipose tissue (eWAT) insulin resistance and increased eWAT CD45+ leukocyte infiltration, compared to floxed littermates [10]. When challenged with a high-fat diet (HFD), *Osmr*^FKO^ mice exhibit systemic insulin resistance and increased eWAT pro-fibrotic and pro-inflammatory gene expression [9]. We have not observed any significant differences in fat mass or food intake in *Osmr*^FKO^ mice on either diet [9,10]. Although our previous data suggest that adipocyte OSM–OSMR signaling may have an important role in the maintenance of WAT immune balance and homeostasis, the mechanisms responsible for this homeostatic role remain unclear.

The alpha chemokine, stromal-derived factor 1 (SDF-1; also known as CXCL12), plays a critical role in hematopoietic stem cell trafficking, homing, and retention, as well as in the accumulation of immune cells positive for the SDF-1 receptor, CXCR4, in injured or inflamed tissues [25]. Recent reports have highlighted the adipose tissue SDF-1–CXCR4 axis in obesity and diabetes. One report postulated that SDF-1 may serve to desensitize adipocytes to insulin [26] and another suggested that SDF-1 modulates angiogenesis in obesity [27]. In addition, a role has been proposed for SDF-1-mediated endothelial cell–stromal interactions, in the maintenance of the physiological adipose tissue cytokine milieu [28]. Direct effects of OSM on SDF-1 expression have been described [29,30,31,32,33], although these studies were not conducted in adipocytes. Here, we provide evidence that adipose tissue SDF-1 levels are affected by the loss of adipocyte OSMR signaling in vivo, and that SDF-1 may have a role in OSM-regulated adipose tissue homeostasis.

## 2. Materials and Methods

### 2.1. Animals and Diets

Male floxed oncostatin M receptor (*Osmr*^fl/fl^) and *Osmr*^FKO^ mice, generated as previously described [9], were obtained from our existing colony. Briefly, *Osmr*^fl/fl^ mice (stock #011081), from Jackson Laboratories, and adiponectin-cre mice, from an in-house colony, were crossed to create the adipocyte-specific OSMR knockout mouse (*Osmr*^FKO^) and littermate floxed controls (*Osmr*^fl/fl^). Mice were housed in a temperature-controlled (22 °C ± 2 °C) and humidity-controlled (45–55%) room under a 12-h light–dark cycle and were allowed ad libitum access to food and water. Mice were fed a high-fat diet (D12451; 45% fat; Research Diets) or a breeder chow diet (Purina LabDiet #5015; LabDiet, St. Louis, MO, USA), as indicated, for 20 weeks, at which time eWAT was collected for gene and protein expression analyses. Mice were 24–26 weeks old when tissues were collected. As previously reported, no differences in body weight or fat mass were observed between genotypes [9,10]. Where indicated, tissue was dissociated into adipocytes and SVCs, as previously described, [9,10] and gene expression analyses were performed as described below. All studies were approved by the Pennington Biomedical Research Center Institutional Animal Care and Use Committee (protocol #961P).

### 2.2. 3T3-L1 Adipocyte Culture and Treatment

Murine 3T3-L1 adipocytes were grown to two days post-confluence and differentiated as previously described [9,10]. Cells were treated for 2–48 h with 0.5 nM OSM (R&D Systems, Minneapolis, MN, USA) or vehicle (0.1% bovine serum albumin in phosphate-buffered saline), as indicated. Media was removed after treatments were completed and saved for analysis. Cell monolayers were washed with PBS and harvested in a buffer containing 150 mM NaCl, 10 mM Tris (pH 7.4) 1 mM EDTA, 1 mM EGTA, 0.5% IGEPAL CA-630, 1% Triton X-100, 1 mM PMSF, 1 µM pepstatin, 50 trypsin inhibitory milliunits of aprotinin, 10 µM leupeptin, 1 mM 1, 10-phenanthroline, 0.2 mM sodium orthovanadate, and 100 µM sodium fluoride or RLT buffer (Qiagen, Germantown, MD, USA), for protein and RNA extraction, respectively. The cell suspensions were subjected to a freeze–thaw cycle at −80 °C, passed through a 20-gauge needle five times, and clarified via centrifugation at 13,000× *g* for 10 min at 4 °C.

### 2.3. Transient Transfection of 3T3-L1 Adipocytes

Fully differentiated murine adipocytes were transiently transfected with si*Osmr* or a non-targeting vector, as previously reported [9]. Briefly, cells were trypsinized, resuspended in DMEM+10% fetal bovine serum without antibiotics, counted, and re-plated into 6-well (protein) or 12-well (RNA) plates at a final concentration of 1.16 × 10^5^ cells per cm^2^. A transfection cocktail containing Dharmafect DUO Transfection Reagent (Dharmacon/Horizon Discovery Group, Lafayette, CO, USA), OptiMEM culture medium (Thermo Fisher, Waltham, MA, USA), and si*Osmr* or non-targeting siRNA (On-Target Plus Smartpool, Dharmacon; final concentration = 100 nM), was then added to the cells. Cells were incubated for 24 h and media were changed to DMEM + 10% FBS without antibiotics. After an additional 24 h, media were changed to DMEM + 1% fetal calf serum without antibiotics, and OSM or vehicle treatments were initiated. Media was removed after treatments were completed and stored for later analyses. Cells were washed with PBS and harvested for protein or RNA extraction.

### 2.4. Gene Expression Analyses

Total RNA was isolated from cell monolayers using the RNeasy Mini Kit (Qiagen) and from eWAT homogenates using the RNeasy Lipid Tissue Mini Kit (Qiagen), as previously described [9,10]. RNA concentrations were quantified using a NanoDrop ND-1000 UV–Vis Spectrophotometer. Reverse transcription was performed using the High-Capacity cDNA Reverse Transcription Kit (Applied Biosystems, Foster City, CA, USA) with 2 µg of RNA. Quantitative PCR was then performed using the SYBR Premix with ROX Plus (Takara Bio, Mountain City, CA, USA) with 4 ng of cDNA and run on the Applied Biosystems 7900HT system with SDS 2.4 software (Applied Biosystems). Thermal cycling conditions were as follows: 2 min at 50 °C, 10 min at 95 °C, 40 cycles of 15 s at 95 °C, and 1 min at 60 °C; dissociation stage: 15 s at 95 °C, 15 s at 60 °C, and 15 s at 95 °C. All target genes were normalized to *Ppia* (peptidyl prolyl isomerase A), *Nono* (non-POU domain containing octamer-binding protein), and *Ubb* (ubiquitin B). The primers used appear in Table 1 below.

### 2.5. Immunoblotting

Protein concentrations of cell lysates, tissue homogenates, and media samples were quantified using the BCA Kit for Protein Determination (Sigma-Aldrich, St. Louis, MO, USA). A total of 50–100 µg protein per well were loaded on 5% or 10% polyacrylamide gels and transferred to 0.45 µm nitrocellulose membranes (Bio-Rad, Hercules, CA, USA). Membranes were probed and imaged using standard immunoblotting techniques, as previously described [9,10,21]. Proteins were detected using a horseradish peroxidase-conjugated secondary antibody (Jackson ImmunoResearch, West Grove, PA, USA) and SuperSignal West Pico PLUS reagents (Thermo Fisher). Anti-STAT5A (E289; ab32043; rabbit monoclonal) and OSMRβ (AF662; goat polyclonal) antibodies were purchased from Abcam and R&D Systems, respectively.

### 2.6. ELISA and Cytokine Arrays

SDF-1 levels in cell culture media were assessed with a Mouse CXCL12/SDF-1 alpha Quantikine ELISA Kit (catalog number MCX120; R&D Systems), according to manufacturer instructions. Cytokine protein expression levels in eWAT (n = 6 per genotype) were assessed using a Proteome Profiler Mouse Cytokine Array Kit, Panel A (catalog number ARY006; R&D Systems), according to manufacturer instructions.

### 2.7. Statistical Analyses

Data were analyzed using GraphPad Prism software (Version 8). Differences between groups were calculated using Student’s t-tests. Results were considered significant when *p* < 0.05.

## 3. Results

We have previously reported insulin resistance and adipose tissue inflammation in *Osmr*^FKO^ mice on high-fat or chow diets [9,10]. Direct effects of OSM signaling on SDF-1 expression have been reported [29,30,31,32,33], and recent publications have highlighted a role for adipose tissue SDF-1 in obesity and metabolic disease [26,27]. Given these previous findings, we sought to examine the effects of a loss of adipocyte OSM signaling on adipose tissue SDF-1 expression, using in vitro and in vivo models.

### 3.1. Effects of In Vivo OSMR Deletion on Adipose Tissue SDF-1 Expression

**Figure 1:**
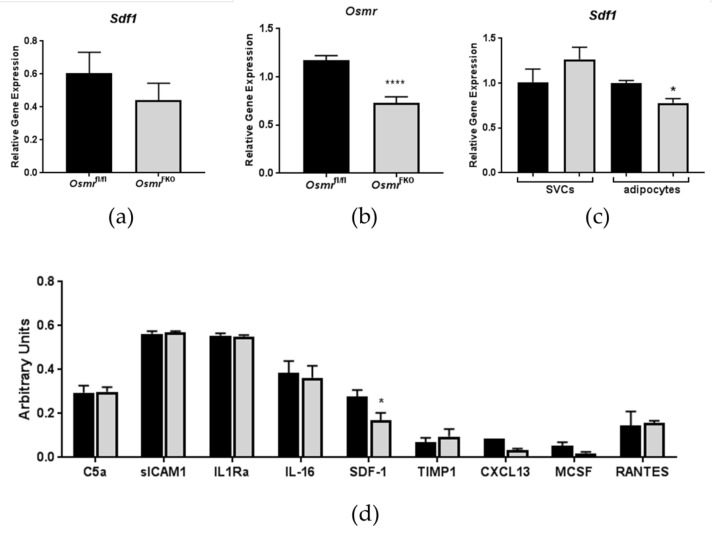
Stromal-derived factor 1 (SDF-1) expression is decreased in epididymal adipose tissue (eWAT) from *Osmr*^FKO^ mice. Gene expression was analyzed in (**a**) eWAT from chow-fed *Osmr*^FKO^ or floxed *Osmr* (control) mice (n = 6–8/genotype). (**b**) *Osmr* knockdown confirmation in eWAT from high fat diet-fed mice (n = 6–8/genotype). (**c**) Fractionated eWAT from high-fat-diet-fed *Osmr*^FKO^ mice (n = 2–3 pooled samples/genotype containing tissue from 2–3 mice per sample) and expression levels were normalized to *Ppia*. (**d**) Protein levels of various cytokines and chemokines were assessed in eWAT from *Osmr*^FKO^ and control mice fed a high-fat diet (n = 6/genotype). Black bars = control (floxed) animals; gray bars = *Osmr*^FKO^. * *p* < 0.05, **** *p* < 0.001 vs. floxed controls.

Interestingly, SDF-1 gene expression was decreased in eWAT from chow-fed *Osmr*^FKO^ mice when compared to floxed controls, although this decrease was not significant (Figure 1a). *Osmr* gene expression was significantly decreased in the eWAT of HFD-fed *Osmr*^FKO^ mice (Figure 1b), thereby confirming the knockout in these mice. In the eWAT of HFD-fed *Osmr*^FKO^ mice that was dissociated into adipocytes and SVCs, *Sdf1* gene expression was significantly lower in the adipocytes, but not the SVCs, of *Osmr*^FKO^ mice (Figure 1c). SDF-1 protein expression levels were also significantly lower in the eWAT of HFD-fed *Osmr*^FKO^ mice when compared to controls (Figure 1d). These results suggest that the regulation of adipose tissue SDF-1 in the *Osmr*^FKO^ mouse occurs, in part, at the level of the adipocyte, and that adipocyte OSMR signaling plays an indirect role in the regulation of SDF-1 expression.

### 3.2. Effects of OSM Administration on SDF-1 Expression in 3T3-L1 Adipocytes

**Figure 2:**
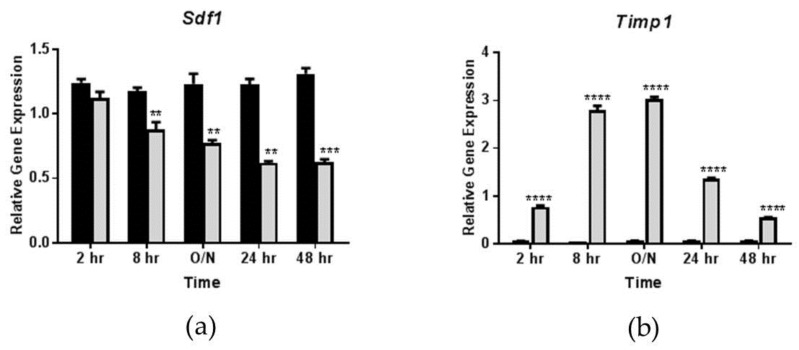
Oncostatin M (OSM) suppresses adipocyte *Sdf1* expression in a time-dependent manner. Fully differentiated 3T3-L1 adipocytes were exposed to 0.5 nM OSM for 2 h, 8 h, ~12 h (overnight; O/N), 24 h, or 48 h. Gene expression was then analyzed and expression levels were normalized to a numerical average of three housekeeping genes (*Ppia*, *Ubb*, and *Nono*). (**a**) Effects of 0.5 nM OSM on *Sdf1* expression in 3T3-L1 adipocytes; (**b**) Effects of OSM on *Timp1* expression in 3T3-L1 adipocytes (shown as a positive control). Black bars = vehicle-treated cells; gray bars = OSM-treated cells. ** *p* < 0.01, *** *p* < 0.001, and **** *p* < 0.0001 vs. vehicle control. This experiment was performed twice.

Previous reports indicate that OSM induces SDF-1 gene and protein expression in various cell types and tissues including mouse heart, mouse mesenchymal stem cells, human cardiomyocytes, and human fibroblasts, but not in adipocytes [29,30,31,32,33]. Since we observed decreased SDF-1 expression in the eWAT of *Osmr*^FKO^ mice, we assessed whether OSM could modulate adipocyte SDF-1 expression. Surprisingly, we found a significant and sustained suppression of *Sdf1* expression by OSM in fully differentiated 3T3-L1 adipocytes. In this study, where cells were treated with OSM for time periods varying from 2–48 h, *Sdf1* expression was significantly decreased by OSM in a time-dependent manner, with a maximal reduction in expression observed at the 24 h time point (Figure 2a). The effects of OSM on the expression of the tissue inhibitor of metalloproteinases 1 (*Timp1*), a known OSM target, were examined as a positive control (Figure 2b).

### 3.3. Effects of OSM Administration on SDF-1 Expression in OSMR-Deficient 3T3-L1 Adipocytes

**Figure 3:**
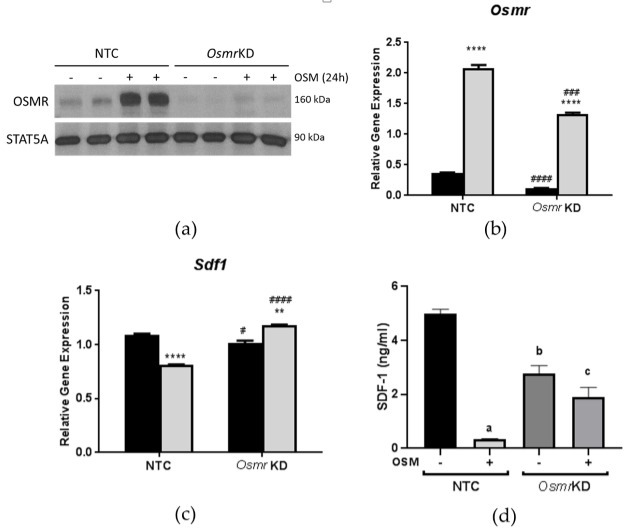
Oncostatin M receptor (OSMR) is required for *Sdf1* suppression in adipocytes. Fully-differentiated 3T3-L1 adipocytes were transfected with si*Osmr* or a non-targeted control siRNA; 48 h after transfection, cells were exposed to 0.5 nM OSM for 24 h. Gene expression was then analyzed and expression levels were normalized to a numerical average of three housekeeping genes (*Ppia*, *Ubb*, and *Nono*). (**a**,**b**) Efficiency of OSMR knockdown. (**c**) The suppressive effect of OSM on *Sdf1* expression is lost with OSMR silencing. (**d**) Effects of OSMR knockdown on SDF-1 secretion into culture media. In panels (**a**,**b**), black bars = vehicle-treated cells; gray bars = OSM-treated cells. KD = knockdown. ** *p* < 0.01 and **** *p* < 0.0001 vs. vehicle controls. # *p* < 0.05, ## *p* < 0.01, and #### *p* < 0.0001 vs. NTC. In panel (**d**), a = *p* < 0.001 vs. NTC; b = *p* < 0.01 vs. NTC; c = *p* < 0.05 vs. NTC + OSM. This experiment was performed twice.

To assess the direct involvement of OSM signaling in decreasing *Sdf1* expression, we knocked down OSMR in 3T3-L1 adipocytes and, subsequently, treated cells with OSM for 24 h. Indeed, OSM-treated cells, transfected with a scrambled siRNA sequence, exhibited the expected diminution in *Sdf1* expression, while cells transfected with si*Osmr* actually exhibited a slight increase in *Sdf1* expression (Figure 3a). Since SDF-1 is a secreted protein, we also assessed its levels in cell culture media from the knockdown experiments. Interestingly, in cells with intact OSMR expression, OSM significantly suppressed SDF-1 secretion. However, in cells lacking OSMR, the suppression of SDF-1 secretion by OSM was blunted (Figure 3b). These results suggest that OSM signaling suppresses adipocyte SDF-1 expression at the gene and protein levels, and that this effect depends on OSMR.

## 4. Discussion

Interactions between adipocytes and SVCs play a role in regulating adipose tissue homeostasis, partly by producing cytokines and chemokines to regulate immune cell balance. We have previously reported that *Osmr*^FKO^ mice, with no differences in body weight or fat mass, exhibit insulin resistance and eWAT inflammation [9,10]. When *Osmr*^FKO^ mice are fed a chow diet, insulin resistance is confined to the eWAT level; but when challenged with a high-fat diet, they develop systemic insulin resistance and increased eWAT inflammation [9,10]. Here, we report that some of these phenotypic effects are associated with altered eWAT SDF-1 gene and protein expression.

Previous studies have demonstrated that OSM induces SDF-1 expression, at both the gene and protein levels, in a variety of cell types [29,30,31,32,33]. In our studies, we did not observe increases in SDF-1 with OSM treatment in vitro; instead, we observed suppression of SDF-1 expression. A very recent study of mouse lung reports decreased SDF-1 gene and protein expression upon administration of an OSM-encoding adenoviral vector [34]. Our current results demonstrate that *Osmr*^FKO^ mice exhibit decreased *Sdf1* gene expression in epididymal adipocytes and decreased SDF-1 protein expression in whole eWAT. In an attempt to further assess the cell autonomous effects of OSM–OSMR signaling on adipocyte SDF-1 expression, we treated 3T3-L1 adipocytes with OSM. We observed that OSM decreased *Sdf1* expression in a time-dependent manner, and that this effect was partly dependent on adipocyte OSMR expression. In 3T3-L1 adipocytes lacking OSMR, SDF-1 gene expression and protein secretion were increased, compared to OSM-treated control cells. However, in eWAT adipocytes from *Osmr*^FKO^ mice, SDF-1 gene expression and protein expression levels were significantly decreased. These contrasting in vitro and in vivo results strongly suggest that the decreased SDF-1 expression in our knockout mice was not due to a direct effect of OSM, but may instead be due to an OSM target gene (or genes) in adipocytes whose regulation is altered by the OSMR knockdown. It is likely that this target gene(s) may alter adipokine production, secretion, and signaling to other adipose tissue stromal cells.

A recent report indicates that adipocyte-specific SDF-1 knockout mice are more insulin-sensitive than their floxed littermates, an effect that may result from the loss of the insulin-desensitizing autocrine actions of adipocyte-derived SDF-1 [26]. In this study, it was suggested that stromal cell-derived SDF-1 is responsible for the classic chemotactic effects of the protein [26]. Another study demonstrated a robust increase of SDF-1 in the WAT of obese mice. The systemic administration of an antagonist to CXCR4 (the SDF-1 receptor) in these mice reduced adipose tissue macrophage accumulation and inflammation, and improved whole-body insulin sensitivity [35]. In that report, the authors concluded that SDF-1 was required for the establishment of obesity-induced adipose tissue inflammation and systemic insulin resistance. In contrast to these reports, we observed increased insulin resistance and eWAT inflammation [9,10] associated with decreased SDF-1 expression (Figure 1) in our *Osmr*^FKO^ mice. These observations support the assertion that the effects on SDF-1 observed in this mouse model are likely an indirect result of alterations in other chemokines and cytokines. Notably, the previous data described here, along with our current data, were obtained from male mice. Given the known in vitro effects of OSM on the estrogen receptor [36], it will be important to conduct similar studies including female mice.

Since we have previously shown that OSM itself is elevated in eWAT in our knockout mice [9,10], the actions of OSM on non-adipocyte cells may be enhancing (or negating) the production of a factor that is altering adipocyte SDF-1 production and secretion. The differences observed in SDF-1 expression between our in vitro and in vivo studies may be attributable to the lack of stromal cells in the in vitro experiments. SDF-1 is produced by several cell types, thus it is possible that adipocyte SDF-1 secretion may act in a paracrine manner to partially regulate adipose stromal cell SDF-1 production. Taken together, our current results, along with previous results from our lab and others, suggest that adipocyte OSM–OSMR signaling may serve as a homeostatic regulator of adipose tissue SDF-1 levels. However, the mechanisms underlying: (1) OSM-induced SDF-1 suppression in adipocytes in vitro; (2) decreased SDF-1 expression in adipose tissue of *Osmr*^FKO^ mice; and (3) whether similar effects occur in female mice, remain unclear. Future studies will focus on elucidating these mechanisms, as they could further contribute to our understanding of how cytokine signaling between SVCs and adipocytes contribute to the maintenance of adipose tissue homeostasis.

## 5. Conclusions

In this study, we demonstrated that OSM suppresses SDF-1 gene expression and protein secretion in cultured adipocytes, and that *Osmr*^FKO^ mice also exhibit decreased adipose tissue SDF-1 gene and protein expression. These results suggest that OSMR mediates decreased adipocyte SDF-1 expression both in vitro and in vivo, but that differing mechanisms govern this regulation. We conclude from these results that adipocyte OSM–OSMR signaling is a regulator of adipose tissue SDF-1 levels.

## Figures and Tables

**Table 1:** Primers used for qPCR.

Gene Name	Forward	Reverse
*Sdf1*/*Cxcl12*	GAGCCAACGTCAAGCATCT	CCACTTTAATTTCGGGTCAATGC
*Timp1*	ACCTGATCCGTCCACAAACA	GGGGTGTGCACAGTGTTTCC
*Ppia*	CCACTGTCGCTTTTCGCCGC	TGCAAACAGCTCGAAGGAGACGC
*Ubb*	CCAGTGGGCAGTGATGG	GCTTACCATGCAACAAAACCT
*Nono*	CATCATCAGCATCACCACCA	TCTTCAGGTCAATAGTCAAGCC
*Osmr*	CGTTCCCCTGTGAGGCCGAG	TCCTCCAAGACTTCGCTTCGGG
